# Reporting of physical activity levels in intensive care unit survivors

**DOI:** 10.1038/s41598-024-83262-1

**Published:** 2025-03-05

**Authors:** Sakshi Surve, Mukesh Kumar Sinha, Vishal Shanbhag, G. Arun Maiya

**Affiliations:** 1 Department of Physiotherapy, Manipal College of Health Professions, Manipal Academy of Higher Education, Manipal, Karnataka 576104 India; 2 Department of Critical Care Medicine, Kasturba Medical College, Manipal, Manipal Academy of Higher Education, Manipal, Karnataka 576104 India; 3 Centre for Podiatry and Diabetic Foot Care and Research, Department of Physiotherapy, Manipal College of Health Professions, Manipal Academy of Higher Education, Manipal, Karnataka 576104 India

**Keywords:** Intensive care units, Critical illness, Intensive care units acquired weakness, Physical activity, Observational study, Public health, Therapeutics

## Abstract

The primary aim of this study was to report the physical activity profiles and the functional status of critically ill patients at one-month post-discharge using the ‘Physical Activity Scale for the Elderly (PASE)’ questionnaire. Study participants included were between 45 and 75 years of age, admitted to ICUs for a minimum of 24 h. Altogether, 110 study participants were included by consecutive sampling, from which six were lost to follow up. This prospective observational study was carried out in the ICU settings of Kasturba Hospital, Manipal. All the participants were assessed for ICU-acquired weakness at ICU discharge and were later followed up after one month for their physical activity level via telephonic follow-up. Participants’ mean age was 57.5 ± 9.82 years, out of which 71% were female. The prevalence of ICU-acquired weakness was found to be 80% in these participants. The median PASE score was 5 (2–27) at one-month follow-up among the participants. Medical Research Council (MRC) sum score showed a statistically significant positive moderate association with PASE score (‘*r* = 0.70, *p* < 0.05). The study showed that about 97.1% of ICU survivors lead a sedentary lifestyle and showed alarmingly high levels of physical inactivity at one month post-hospital discharge.

## Introduction

Advancements in critical care medicine have steered a steady rise in the survival rates of the critically ill in the past few years^1–3^. These patients are termed as ‘ICU survivors. ICU-acquired weakness (ICU-AW) is a common condition in patients admitted to the intensive care unit (ICU). It is characterized by weakness in the muscles due to prolonged immobility, critical illness, and other factors related to intensive care settings. ICU-AW can significantly impact recovery and quality of life following hospitalization. However, this has also increased the risk of susceptibility to serious complications of prolonged ICU stay like ‘Intensive Care Unit acquired weakness’ (ICU-AW) and ‘Post Intensive Care Syndrome’ (PICS)^4^.

There are several subtypes of ICU-AW, including critical illness polyneuropathy (CIP) critical illness myopathy (CIM), and ‘Critical Illness Neuromyopathy’ (CINM)^5^.

ICU-AW is a medical diagnosis based on physical examination of muscle strength tests, primarily the ‘Medical Research Council’ (MRC) sum-score and handgrip strength assessment using a handheld dynamometer^4^. Apart from this, other electrophysiological assessment methods such as muscle ultrasound, electromyography (EMG), and muscle biopsy can be alternatives in unconscious, uncooperative patients^6^. In summary, ICU-AW is ‘a clinically detected condition characterized by diffuse, symmetric weakness involving the limbs and respiratory muscles. Consistent findings show subjective weakness and reduced exercise capacity compared to pre-ICU admission reported in ICU survivors^7^. It was seen that all ICU survivors reported poor function post-ICU discharge and accredited this to muscle mass deficit, proximal girdle muscle weakness, and fatigue. As a result, ICU survivors often have reported reduced physical activity levels in the post-ICU discharge period^8^.

Physical activity has been defined as ‘any bodily movement produced by skeletal muscles that requires energy expenditure. Physical activity refers to all movements, including during leisure time, for transport to and from places, or as part of a person’s work’^9^. Since physical function is only partially recovered after ICU discharge, assessing it during and after an ICU stay can be crucial in identifying patients at a significant risk of deteriorating physical outcomes, planning appropriate intervention strategies, and mapping recovery trajectories^10^. As a result, quantifying the physical activity levels among the ICU survivors post-ICU discharge needs further exploration. Literature regarding the post-ICU/hospital phase is inadequate; thus, despite lingering deficits, the current literature provides only a partial understanding of the physical activity status of the patients during this phase. Various screening tools and questionnaires can be used to screen physical activity levels. The ‘Physical Activity Scale for the Elderly’ (PASE) questionnaire was developed to evaluate lifestyle physical activity in older adults over 7 days^11^.

The purpose of this study was to report the physical activity levels and functional status among ICU survivors at one month post-discharge using a PASE questionnaire, which would help formulate a better prognosis and home-based rehabilitation for the therapists.

## Materials and methods

This observational study aimed to evaluate and report the physical activity levels of ICU survivors post-discharge from intensive care units. The study adhered to relevant guidelines and regulations, specifically following the Strengthening the Reporting of Observational Studies in Epidemiology (STROBE) Statement. The research was conducted at Kasturba Hospital, Manipal. A sample size of 144 participants was determined, accounting for a potential 20% non-response rate. All necessary ethical approvals were obtained from the Ethics Committee at KMC & KH [IEC2: 211/2023]. The study was registered with the Clinical Trials Registry India (CTRI) under the registration number CTRI/2023/08/056404. Informed consent was acquired from all participants before their recruitment into the study.

Participants were individuals between 45 and 75 years of age, a minimum of 24 h of ICU admission with a physiotherapy referral. Exclusion criteria comprised of patients with a GCS score of less than 12, any cognitive impairment before or during ICU stay, any fracture limiting mobility, patients undergoing any cancer therapy or any renal replacement therapy in the last six months or with any history of renal transplants, history of any neurological impairment or currently admitted to ICU for neurological conditions, readmissions to the ICU, pregnant women. We employed a consecutive sampling method to select participants admitted to the Intensive Care Unit (ICU) at Kasturba Hospital, Manipal. This approach involved enrolling patients aged 45 to 75 years and a minimum of 24 h of ICU admission with a physiotherapy referral. By using consecutive sampling, we aimed to minimize selection bias and ensure that all eligible patients were given an equal opportunity to participate in the study. The estimated sample size was 144, with the level of significance set at 1.96 and an adjustment of the non-response rate at 20%.

Patients were screened for eligibility criteria, and written informed permission was acquired for eligible patients. Demographic details of the patient, disease status, and information regarding current medications and ongoing physiotherapy treatment were obtained.

Along with the demographics, the ‘Duke Activity Status Index’ (DASI) and ‘Charlson Comorbidity Index’ (CCI) scores were also assessed^12,13^. Patients were then evaluated for their muscle strength on the day of their ICU discharge, using ‘The Medical Research Council (MRC) sum score,’ which includes mainly six significant groups of muscles, namely ‘shoulder abductors, elbow flexors, wrist extensors, hip flexors, knee extensors, and ankle dorsiflexion.’ The muscle strength for the above muscle groups was scored from 0 to 5, 0 indicating no apparent or palpable muscle contraction and 5 indicating the complete range of motion against gravity at maximal resistance^14,15^. A score lesser than 48 from total score of 60 is an indicator of clinical diagnosis of ICU-AW^16^.

Also, the patient’s functional status on the day of ICU discharge was evaluated using the ‘Functional Status Scale in the Intensive Care Unit’ (FSS-ICU). Each component is rated on an 8-point ordinal scale from 0 to 7^17^.

**Fig. 1:**
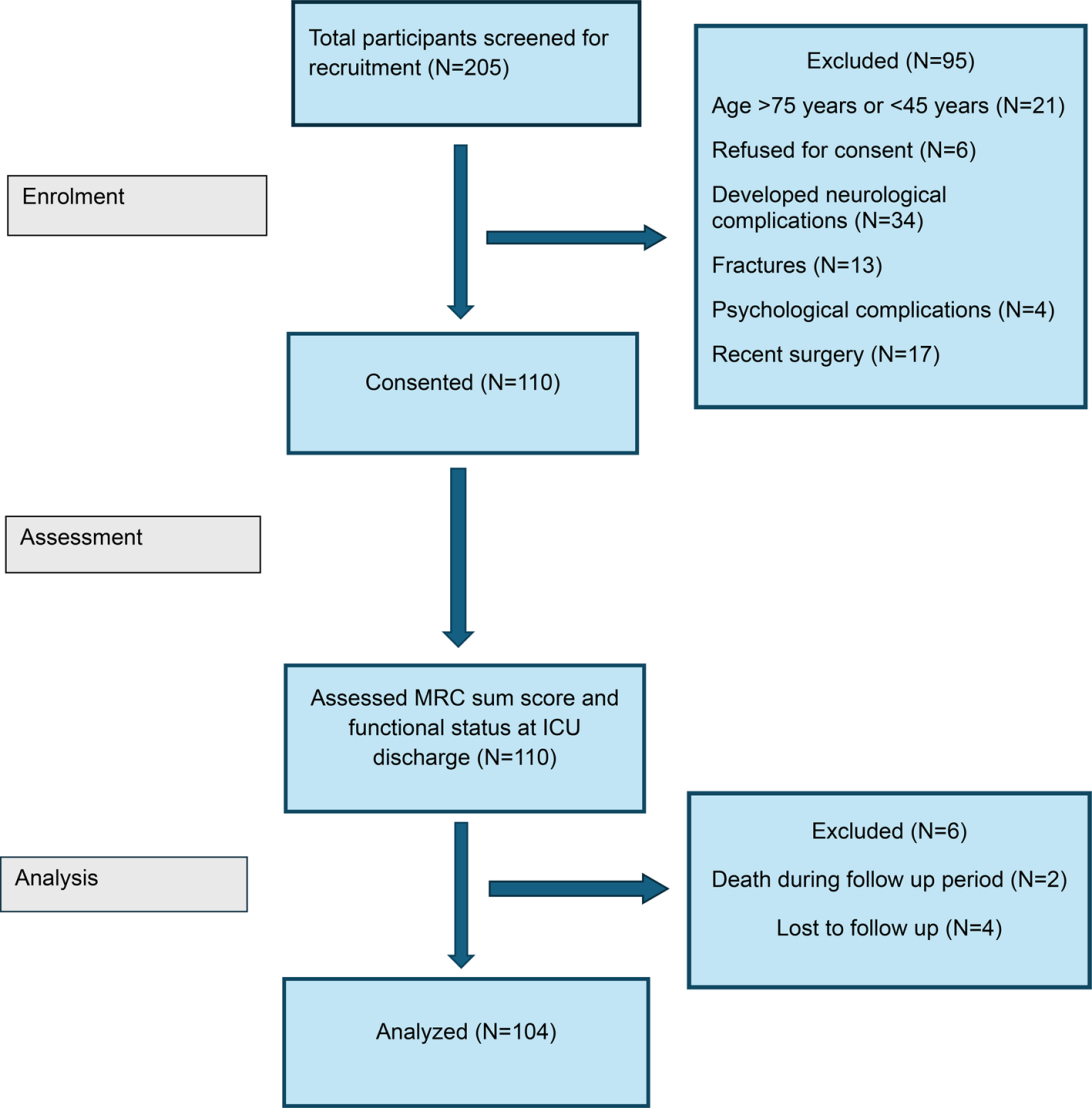
Participants flow through the study.

The second point of patient assessment was at one-month intervals post-ICU discharge. At this point, patients were assessed for their physical activity levels using the ‘Physical Activity Screening for the Elderly (PASE)’ questionnaire over a telephonic follow-up. A pilot test was conducted with a small group of four ICU survivors to assess the clarity of the questionnaire and identify challenges in capturing their physical activity levels after discharge. Feedback from the participants was incorporated, and the feasibility of conducting interviews over the phone was evaluated. This process ensured that the questionnaire accurately reflects the physical activities that ICU survivors are likely to engage in one-month post-discharge. The flow of participants at various checkpoints has been elaborated in Fig. 1.

### Data analysis

The data was analyzed using Jamovi software (version 2.4.14). Demographic data was described using descriptive statistics and mean and median values of the MRC sum score and PASE score. Normality was checked using the Shapiro-Wilk test, which showed the data to be normally distributed. The data was analyzed using Jamovi (version 2.4.14) software. The correlation of mean scores of MRC sum score, DASI score, and FSS-ICU score, and mean duration of mechanical ventilation at ICU discharge with PASE score at one-month post-ICU discharge was done using Spearman’s rank correlation test.

### Clinical significance

From the above results, our study concluded that ICU survivors have exceptionally poor levels of physical activity, thus giving rise to developing and incorporating strategies for well-defined, individualized, and early rehabilitation protocols for ICU survivors post-discharge to prevent future complications. Based on the observations made in this study, there is a need for further exploration of intervention studies for the rehabilitation of critical illness survivors.

## Results

Two hundred and five participants had been screened for eligibility for participation in the study. A total sum of 110 participants (34 males and 76 females) had been included, out of which four were lost to follow-up and two were deceased. Participant demographics have been summarized in Table 1.

**Table 1:** Baseline attributes of study participants.

Demographics	For males (*N* = 30)	For females (*N* = 74)	Total (*N* = 104)
Age, mean (SD)	59.5 ± 9.59	56.8 ± 9.87	57.5 years (9.82)
BMI, mean (SD)	23 ± 2.25	24.3 ± 3.76	24 (3.44)
Active smoker (%)	8 (7.7%)	1 (1%)	8.7%
CCI, median (IQR)	4 (2–5)	2 (1–4)	2.89 (212)
Duration of mechanical ventilation, mean (SD)	38.9 ± 21	39.6 ± 21.6	39.4 h (21.3)
Length of stay in the ICU, mean (SD)	4.8 ± 2.77	4.2 ± 1.83	4.41 days (214)
Length of stay in hospital, mean (SD)	5.9 ± 3.18	5.4 ± 2.24	5.6 days (2.54)

BMI = Body Mass Index, CCI = Charlson Comorbidity Index, IQR = interquartile range, ICU = intensive care unit.

A large proportion of participants (78%) had developed at least one or more than one comorbidity a priori to ICU admission. They were grouped as participants with no co-morbidities (22%), participants with 1–2 comorbidities (47%), and participants with three or more co-morbidities (31%).

Table 2 summarizes the prevalence of ICU-acquired weakness amongst the participants, both in males and females, at ICU discharge.

**Table 2:** Prevalence of ICU-acquired weakness in the participants.

MRC sum score	Prevalence in males % (*N* = 30)	Prevalence in females % (*N* = 74)	Total prevalence % (*N* = 104)
(less than 48) [indicator of ICU-AW]	22 (21.2%)	61 (58.7%)	83 (79.8%)

MRC = Medical research council, ICU-AW = intensive care unit-acquired weakness.

The average DASI score for the participants was 8.17 (SD = 4.46), whereas the average FSS-ICU score was 22.1(SD = 6.12). Table 2 summarizes the prevalence of ICU-acquired weakness based on the MRC sum score.

We found the median total PASE score was 5 (2–27), which indicates that ICU survivors have a very sedentary lifestyle one month after ICU discharge. Males were observed to be more physically active, with a median PASE score of 9 (5–27), than females, with a median score of 5 (2–25).

The PASE scores exhibited a statistically significant positive moderate correlation with the Medical Research Council (MRC) sum score taken at ICU discharge (*r* = 0.57, *p* < 0.05). The moderate correlation suggests a meaningful relationship, implying that patients with better muscle strength are likely to engage in more physical activity post-discharge.

Additionally, we found a statistically significant positive high correlation between the DASI (Duke Activity Status Index) score obtained at the time of ICU discharge and the PASE scores measured one month later (*r* = 0.64, *p* < 0.05). This strong correlation suggests that patients who reported higher functional capacity and ability to perform daily activities, as indicated by the DASI score, were also likely to maintain higher levels of physical activity after leaving the ICU.

Furthermore, there was a statistically significant positive moderate correlation between the PASE scores and the FSS-ICU (Functional Status Score for the ICU) scores at discharge (*r* = 0.59, *p* < 0.05). This relationship indicates the importance of early interventions that facilitate functional improvement in the ICU, which may lead to better long-term activity levels post-discharge.

## Discussion

The purpose of this study was to report patients’ physical activity levels and functional status during ICU discharge, post-ICU hospital stay, and post-hospital discharge. This is critical against the backdrop of post-essential complications of illness, such as ICU-acquired weakness which may impact the physical function of ICU survivors.

This study used a PASE questionnaire to report the physical activity levels in ICU survivors as they transition from the ICU and acute care hospital settings into the community settings. We found that physical activity levels were very low at one-month post-discharge, as seen from the mean PASE score values for both males and females, suggesting substantial limitations in the functional status of the patients physically. This may be explained by several environmental factors, such as settings about space constraints within the surroundings, family responsibilities, transport to and from the rehabilitation settings, financial conditions, and accessibility. Another likely explanation for the above results is the patient-related factors such as fatigue and pain, mental disturbances, and drowsiness. In the Indian context, beliefs such as ‘recovery post critical illness require complete rest,’ and societal structure with large, joint families that can care for the patient could also play a significant role in the limited physical activity of the ICU survivors.

The observed gender imbalance in this study, with 71% of participants being female, calls for a more thorough investigation into its possible impact on our findings. It is vital to assess how this disparity may affect the generalizability of the results, especially considering that previous research has indicated a higher prevalence and severity of ICU-Acquired Weakness (ICU-AW) in females. Several factors could contribute to this trend. Biological differences, such as variations in muscle mass and hormonal influences, may make females more susceptible to developing severe ICU-AW. Additionally, social factors, including differing perceptions of disease and health-seeking behaviors, might shape the demographics of ICU admissions and the subsequent development of ICU-AW.

Taking these factors into account, the significant representation of female participants (71%) in our study could enhance findings related to the pathophysiology and progression of ICU-AW, potentially leading to increased recognition of gender-specific risks and outcomes. However, this imbalance also represents a limitation, underscoring the importance of caution when extrapolating these results to the wider population. Future research should strive to address this gap by ensuring a more balanced inclusion of male participants, thereby promoting a comprehensive understanding of how gender dynamics influence ICU-AW outcomes.

PASE scores at one-month post-ICU discharge were moderately correlated to the MRC sum score assessed on the day of ICU discharge (*r* = 0.57, *p* < 0.05). This finding may suggest that the presence or absence of ICU-acquired weakness at the time of ICU discharge could be one of the key determinants dictating the physical activity levels post-ICU discharge. Interestingly, in our study we found a high incidence of about 79.8% of participants who had sum score lesser than 48 from 60 indicating presence of ICU-AW^16^. Additionally, we also noted that 12.5% of the participants had shown severe weakness, as indicated by an MRC sum score of below 36^18^. However, it must also be noted that our study sample represented a female population more than males. This can have an impact on the incidence rates of ICU-acquired weakness as existing evidence suggests female sex is one of the risk factors for developing ICU-acquired weakness^16^.

The duration of mechanical ventilation was correlated negatively (*r*= -0.29, *p* = 0.002) with the PASE score at the one-month follow-up. The already known fact could explain that prolonged mechanical ventilation duration has been shown to increase the risk of disuse atrophy and various other secondary complications, which may eventually prolong the time span of ICU stay and, consequently, the recovery period post-discharge^19,20^.

However, in our study, other confounding factors such as varied treatment practices, duration and dosage of sedation, day of ICU discharge, existing co-morbidities and primary disease, and ongoing medications were not considered and could have impacted the results.

The risk of ICU-acquired weakness (ICUAW) is shaped by various environmental factors and underlying health conditions. Active rehabilitation and a multidisciplinary approach can help mitigate the risks associated with prolonged immobilization, while policies that encourage early mobilization tend to result in better patient outcomes. After discharge, successful integration into home care and community rehabilitation is crucial for enhancing recovery. Comorbidities, including diabetes and cardiovascular disease, increase a patient’s vulnerability to both ICUAW and decreased physical activity. Sociodemographic factors, such as gender and socioeconomic status, also impact health outcomes and the generalizability of research findings. Furthermore, psychological aspects like anxiety, depression, and motivation play a significant role in the recovery process. These factors can greatly influence recovery trajectories, highlighting the complexity of recovering from ICUAW and underscoring the necessity for comprehensive assessments in future research.

Based on CCI score, our study noted that patients were moderately unwell at ICU admission. It is well known that co-morbidities were associated with less engagement in physical activity^21^. Our study settings practiced the early mobility approach for ICU-admitted patients. Evidence suggests that although early mobilization did not have a role in increasing the MRC sum score at hospital discharge, it did lead to a fall in the incidence of ICU-AW along with a rise in the number of ventilator free days^22^. There are studies showing that medications such as corticosteroids can have deleterious effects on neuromuscular function^23,24^. This decline in neuromuscular function may also influence the post-discharge physical activity levels.

Similar studies have used accelerometers and reported physical activity in ICU survivors through step counts as they transit from the ICU into the wards or the community settings^25,26^. Unlike our study, which consisted of a relatively younger population, these studies focused on older adults. Contrastingly, our study noted poor physical activity levels despite the lower mean age of the participants. Factors such as occupational status, socioeconomic status, familial responsibilities, sex, and existing co-morbidities could have affected these findings. Interestingly, they found that the physical activity levels did not significantly change patients during the shift from the ICU to the wards. Still, the physical activity levels did show a decline at one-month follow-up in the community settings, as suggested by the average step count of.

the study participants was significantly low^25–27^. These studies found a fair, positive correlation between the step counts and the PASE scores of the participants. Since feasibility and accessibility would pose a major challenge with the study setting being a semi-rural area, accelerometers were not utilized in our study as an estimate of the physical activity of the participants.

Additionally, recent evidence suggests that systemic inflammatory markers, such as the neutrophil-to-lymphocyte ratio (NLR) and platelet-to-lymphocyte ratio (PLR), may serve as significant prognostic indicators. Elevated levels of NLR and PLR are linked to unfavorable outcomes in infectious diseases, cancers, and inflammatory disorders^28^. In ICU survivors, exploring the relationship between physical activity and these inflammatory markers could provide valuable insights into recovery processes. Therefore, future studies could explore the various inflammatory markers and PA trajectory.

Artificial intelligence (AI), also known as machine intelligence, focuses on developing systems capable of performing tasks autonomously. Given the current shortage of healthcare professionals, harnessing technology provides a practical solution. AI has already demonstrated its value in the medical field. One significant application of AI is in monitoring physical activity levels among survivors of Intensive Care Units (ICUs)^29^. These patients often experience considerable physical decline during their hospital stays, which can adversely affect their long-term recovery. By employing AI-driven tools to track activity during and after ICU admission, healthcare providers can obtain valuable insights into the rehabilitation progress of these patients.

## Limitation

The current study has many limitations. Survivors of intensive care units (ICUs) may encounter recall bias when reporting their physical activity levels. Participants often struggle to remember and articulate their activity accurately. Furthermore, the reliance on self-reported data can compromise the accuracy of physical activity measurements. A follow-up period of only one month is unlikely to fully capture the breadth of recovery or changes in physical activity levels; therefore, longer-term follow-ups could offer a more comprehensive perspective on physical activity trends. Acknowledging these limitations underscores the necessity for improved strategies to enhance the reliability of telephonic assessments. The use of standardized scripts and interviewer training can significantly boost the consistency and accuracy of data collection. Additionally, demographic factors, such as age and socioeconomic status, may influence the feasibility and effectiveness of telephonic follow-ups.

## Conclusion

Our study evaluated and reported the physical activity levels among ICU survivors after one month of ICU discharge. However, the one-month time point is crucial as it allows sufficient time for patient recovery and can direct the action plan for designing and implementing rehabilitation protocols for ICU survivors. We recognize that the outcome measures used for assessing physical activity levels were subjective, which may affect the uniformity and generalizability of the results.

## Data Availability

Research data will be available from the corresponding author with a reasonable request.
